# “The Most High-Risk People Are Given the Most High-Risk Drugs in the Most High-Risk Way”: Experiences of Treating Problematic Over-the-Counter and Prescription-Only Medication Use in Substance Misuse Services

**DOI:** 10.3390/pharmacy14040094

**Published:** 2026-06-27

**Authors:** Rosalind Gittins, Roya Vaziri, Ian Maidment

**Affiliations:** 1 Aston Pharmacy School, Aston University, Birmingham B4 7ET, UK; 2 Waythrough, Inspiration House, Unit 22, Bowburn North Industrial Estate, Durham DH6 5PF, UK

**Keywords:** over the counter drug misuse, prescription drug misuse, interviews, staff, substance misuse services

## Abstract

Misuse of over-the-counter (OTC) and prescription-only medicines (POMs) is increasingly recognised as a public health and medicine-safety concern. Although specialist substance misuse services (SMS) increasingly support people affected by OTC/POM misuse, little is known about how SMS staff perceive the characteristics, challenges, and treatment needs of this population. This study explored the experiences of SMS staff to address this evidence gap and inform pharmacy practice and service development. Confidential semi-structured interviews were conducted with staff across five community adult English SMS. Audio recordings were transcribed verbatim and analysed thematically using NVivo^®^. Twenty interviews with varied professionals achieved data saturation. Three overarching themes emerged: (1) characteristics of OTC/POM misuse; (2) staff-perceived patterns among people who misuse OTC/POM; and (3) negative experiences and concerns. Dependence on orally administered opioids (particularly codeine-containing products), benzodiazepines and gabapentinoids predominated. Polypharmacy including illicit substance use was also reported. Withdrawal symptoms frequently perpetuated misuse, and abrupt supply cessation created additional risks. Routine enquiry about OTC/POM misuse and provision of tailored harm-reduction interventions are essential. The findings suggest that pharmacists may have an important role in early identification of problematic OTC/POM use, harm-reduction interventions, medicine review and facilitating referral into appropriate treatment pathways. Further research should examine whether dedicated OTC/POM pathways are required and explore differences in demographic and treatment needs across medicine types.

## 1. Introduction

Misuse of over-the-counter (OTC) and prescription-only medicines (POMs), defined as intentional use outside of clinical or manufacturer guidance, represents a growing challenge for medicine safety and public health [1,2]. Although such misuse accounts for a minority of presentations to specialist substance misuse services (SMS) in England, national datasets may underestimate its true prevalence due to limitations in reporting structures and coding practices [2,3,4]. Pharmacists, as accessible medicine experts, are often the first professionals to observe emerging patterns of problematic use, yet their insights are not routinely captured within SMS data systems.

There is increasing recognition that people who misuse OTC/POM products may have unmet treatment needs. Many individuals perceive SMS as services designed primarily for illicit substance use and may avoid engagement due to stigma, concerns about identity, or a mismatch between service design and their expectations [5,6]. Uncertainty surrounding appropriate interventions, particularly deprescribing, tapering regimens, and substitute prescribing, further complicates care. This is exacerbated by variable commissioning arrangements, limited national guidance, and a sparse evidence base for managing dependence for various commonly misused medicines [7].

OTC/POM misuse is associated with significant physical, psychological, and socio-economic harms, including deterioration in mental health, relationship breakdown, employment instability, and housing insecurity [8,9]. These harms extend beyond the individual, affecting families, communities, and wider health and social care systems [9,10]. OTC/POM misuse also continues to be implicated in drug-related deaths [11], and pharmacists frequently encounter the consequences of such misuse, including drug-related morbidity, toxicity from combination products, and escalating patterns of self-medication. National reports have increasingly highlighted the need for improved identification, safer supply, and more effective interventions across pharmacy and SMS settings [7,12,13].

Despite this, there remains a notable lack of research exploring the experiences of SMS staff, particularly those working in third-sector organisations, who support people misusing OTC/POM [14,15]. Previous work has been limited by the under-representation of staff who work in the third (charity) sector, and those who support individuals who use multiple substances [16,17]. SMS staff occupy a unique position within the treatment system, offering valuable insights into medicine-related behaviours, prescribing challenges, and opportunities for enhanced pharmacy involvement in care pathways.

Understanding their experiences is essential, particularly in the context of workforce pressures, recruitment and retention challenges, and the emotional burden associated with supporting people with complex needs [12,18,19]. Such insights can inform how pharmacy and SMS teams are supported, trained, and integrated into multidisciplinary approaches to medicine optimisation, harm reduction, and safer prescribing.

Therefore, this study aimed to explore the experiences and perceptions of staff working within third-sector community adult SMS regarding the identification, management, and treatment of adults who misuse OTC/POM: the objectives were to identify perceived implications for pharmacy practice and future service development.

## 2. Materials and Methods

A qualitative design using semi-structured interviews was adopted to explore the experiences of SMS staff supporting adults who misuse OTC/POM. This approach has been widely used in research examining medicine misuse and SMS practice and is well-suited to capturing the complexity of medicine-related behaviours [10,16,17,20,21]. Participants were recruited from five community adult SMS delivered by one of the largest third-sector providers in England.

Eligible staff were required to be currently employed in a ‘front-line’ role and have experience supporting adults misusing OTC/POM. All staff met organisational requirements for English proficiency and were aged ≥18 years. Individuals known personally to the interviewer were excluded.

To ensure a broad range of perspectives, SMS managers circulated study information to all eligible front-line staff across their services. Staff who were interested in taking part contacted the lead researcher (RG) directly. Recruitment continued until no substantially new concepts were identified within successive interviews, and therefore data saturation was judged to have been achieved: this was further assessed during concurrent data analysis, with no new codes identified in the final three interviews [22]. Data collection took place between October 2020 and January 2021 during the COVID-19 pandemic. Consequently, interviews were conducted face-to-face, by telephone, or via Microsoft Teams according to participant preference and prevailing public health restrictions.

Ethical approval was obtained from the SMS provider and Aston University’s Life and Health Sciences Ethics Committees (ID#1655). Participation was voluntary, with no incentives offered. All the participants received written information and provided informed consent prior to the interview.

An interview guide was developed from the literature and reviewed by researchers with expertise in pharmacy and SMS, alongside individuals with lived experience and SMS staff, to ensure clarity and relevance: this was used to support consistency across interviews. All the interviews were digitally audio-recorded. Recordings were transcribed verbatim by an independent transcriber, and all identifying information was removed. Each participant was assigned a unique code.

NVivo^®^ (version 12) was used to support data management [23]. Thematic analysis followed Braun and Clarke’s established framework [24]. Interviewing and initial coding were undertaken by RG, with IM verifying coding decisions and RV available to resolve discrepancies. The analysis included active searching for disconfirming evidence to enhance rigour [25]. All qualitative data contributed by the participants were included in the final analysis. The study was conducted and reported in accordance with the Consolidated Criteria for Reporting Qualitative Research (COREQ) checklist [26].

## 3. Results

**Figure 1:**
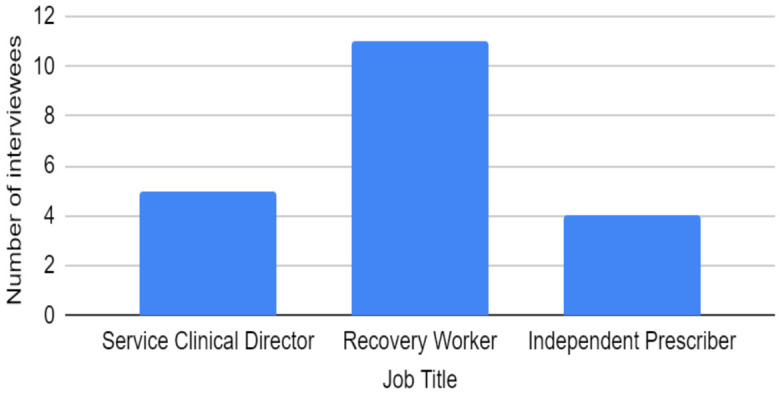
Summary of participant job titles.

Twenty interviews were conducted, lasting a mean of 18 min (range: 7–40 min; Table 1). Recruitment and scheduling were challenging due to pandemic-related pressures and high clinical workloads. Six interviews were conducted face-to-face, thirteen by telephone, and one via Microsoft Teams (Table 1); the participants had a variety of roles (Figure 1).

The participants represented a range of roles across five community SMS, including clinical directors, independent prescribers, and recovery workers. Most were female (60%), and experience in SMS ranged from 14 months to 30 years. Although the participants were not asked, four chose to disclose personal lived experience of OTC/POM misuse, offering additional insight into medicine-related behaviours.

Three overarching themes were identified:

### 3.1. Characteristics of OTC/POM Misuse

The participants predominantly supported individuals misusing opioids (especially codeine-containing products), benzodiazepines, and gabapentinoids, typically administered orally. Medicines were sourced through multiple routes including friends/family, GPs, private prescribers, hospitals, street dealers, the dark web, social media sites, community and online pharmacies. Staff highlighted inconsistent access to drug testing for specific OTC/POM, limiting their ability to identify misuse and tailor harm-reduction interventions.

Patterns of use included daily dependent consumption, escalation in response to psychological stress, and increasing use of multiple suppliers, particularly among those with iatrogenic dependence. Some participants noted variance in response to psychological stress, ease and awareness of availability.

*“Throughout the day… in handfuls… every so many hours they’ll be taking it. Closer and closer together, depending on their stress level… because they couldn’t manage the day to day”*

#016

### 3.2. Staff-Perceived Patterns Among People Who Misuse OTC/POM

The participants commonly described two broad patterns among people presenting with OTC/POM misuse: those who primarily misused legally sourced OTC/POM and those who used OTC/POM alongside illicit substances. The latter commonly used benzodiazepines or opioids to potentiate or counteract the effects of illicit drugs. OTC/POM misuse sometimes preceded transition to illicit opioids, highlighting a medicines-to-illicit-drug trajectory. These categories reflect staff perceptions and were not formally examined within this study.

Those who did not use illicit substances concomitantly were typically younger, female, had no history of illicit drug use, presented with iatrogenic dependence, misused only OTC codeine products, and were described as ‘working-professional types’, who could more readily afford the cost associated with OTC supplies. They were perceived as methodical in obtaining supplies and less likely to identify with SMS. The participants reported that these individuals often required more intensive psychological support and found detoxification particularly challenging.

*“Most of the people… women… when they come off it, they are the ones who struggle… especially young women… emotionally they are struggling… requires more work and psychological principles”*

#014

Views differed on whether separate SMS pathways for OTC/POM misuse should be developed: some felt it could reduce stigma and improve engagement, while others cautioned against reinforcing divisions between types of substance use.

*“I don’t think separating people into different categories is the right thing to do. But… there’s a group of people that would not entertain going to a group where there’s people that do heroin, because they see themselves different…”*

#015

### 3.3. Negative Experiences and Concerns, Comprising Six Sub-Themes

- Limited awareness of harm: The participants reported that many individuals underestimated the risks associated with OTC/POM due to their legal status, ease of availability from pharmacies, or initial therapeutic use. Long-term misuse was often normalised, particularly among older adults with comorbidities who are at increasing risk of harm.

*“A lot of our service users are getting a bit older and they’re a lot of the mindset ‘oh, I’ve been doing this for years—I’ve got a really high tolerance. I can do a lot of stuff with no risk of harm’ but you’re probably more at risk now than you were previously, because you now have other issues with your health”*

#015

- Unmanaged health conditions: OTC/POM were frequently used to self-manage untreated pain or psychological distress. The participants described barriers to accessing appropriate healthcare likely aggravated by COVID-19, including long waiting lists, and reluctance from other services to treat individuals with co-existing substance use. SMS staff expressed concern about becoming a ‘default’ service for complex polypharmacy and health and social care needs.

*“Complex people with multiple medical conditions and complex psychiatric conditions and substance misuse issues… the most high-risk people are given the most high-risk drugs in the most high-risk way…. There is a huge reservoir of people who are practically impossible to treat… ‘heart-sink’ patients with multiple ailments, polypharmacy… [SMS] become a dumping ground for prescribing blunders”*

#001

- Withdrawal symptoms: Withdrawal symptoms were a major driver of continued misuse. The participants described challenges in managing withdrawal safely, particularly when individuals used OTC/POM to self-treat missed doses of substitute medication or withdrawal from illicit substances. The need for increased vigilance was described when prescribing symptomatic relief during detoxification.

*“When it comes to detox time, we’ve got to be really, really careful of what we prescribe because I feel that a lot of ‘medication clients’ enjoy benzos and zopiclone… we should be really wary of that”*

#016

- Adverse events: Significant harms were reported, including gastrointestinal bleeding, renal impairment, paracetamol toxicity, and fatalities, especially associated with high-dose combination codeine products. Concerns were heightened when medicines were sourced illicitly, greater amounts were consumed or used alongside opioid substitute treatment or illicit substances. This led to the participants emphasising the need for repeated and greater prioritisation of harm-reduction measures such as liver function monitoring, advising on cold-water extraction, dose reduction regimens, drug interactions and take-home naloxone provision.

*“Appallingly underweight, with appalling internal bleeding, bulimia, kidney damage, relentlessly pounding themselves to death with Nurofen Plus^®^… ingesting far larger [paracetamol] amounts than the liver is supposed to be able to handle… we have had some deaths… you wipe your liver out”*

#001

- Problematic behaviours: The participants described behaviours associated with dependence, including dishonesty, repeated requests for early prescriptions, spending notable time and money on travelling to different pharmacies, and use of third parties to obtain supplies. Stigma and fear of consequences (e.g., child-protection concerns) contributed to non-disclosure. Constructive challenge and strong therapeutic rapport were viewed as essential.

*“I will challenge… they can relate to what I’m saying… they become more open to telling the truth”*

#016

- Treatment pathways: A lack of clear, consistent pathways for OTC/POM misuse was a major concern. The participants highlighted variability in prescribing practices, limited guidance for tapering regimens, and inconsistent commissioning arrangements. Increased contact frequency, continuity of staff, and blended in-person/remote models developed during COVID-19 were viewed as beneficial. Improved communication with primary care and pharmacy teams was identified as critical for safer medicine management.

*“Either there isn’t a clear pathway or they are not aware of a clear pathway… Having a consistent approach… [commissioning] consistency would help”*

#002

## 4. Discussion

To our knowledge, this is the first study to explore the views of English third-sector SMS staff who are supporting adults who misuse OTC/POM. It highlights potential implications for pharmacy practice, medicine optimisation, and harm-reduction policy. The participants consistently reported oral misuse of codeine-containing products, benzodiazepines, and gabapentinoids, reflecting national surveillance data and previous research: the patterns of dependent, high-frequency use align with established evidence on the trajectory of pharmaceutical misuse [2,3,10,13,27].

The wide range of supply routes, including community and online pharmacies, highlights the central role of pharmacists in early identification, safer supply, and pharmacovigilance; additionally, drug testing could be used as a harm reduction intervention to raise awareness of potential harms when products are being illicitly sourced and have unknown contents [28,29].

The reported use of OTC/POM polypharmacy and potentiation or counteracting of illicit substances reinforces the existing literature and underscores the need for SMS and other healthcare staff, including pharmacy teams, to be proficient at recognising such behaviours [2,10]. These findings also emphasise the importance of prioritising harm reduction interventions and the need to maximise take-home naloxone provision [30,31].

The participants’ accounts of escalating use in response to psychological stress echo the findings of Bagusat et al. that resilience-building and tailored psychosocial interventions may reduce reliance on medicines for emotional regulation [32]. This also aligns with national guidance, which highlights that psychosocial interventions remain the mainstay of effective treatment [7]. Similarly, reports of iatrogenic dependence and self-medication with OTC/POM for underlying physical and mental health needs are also well-established and highlight the importance of people being able to promptly access holistic support, including via pharmacies [33,34].

Concerns about side-effects and toxicity, and particularly with combination codeine products, mirrored risks documented in the pharmacy and toxicology literature, and especially in older age, where high doses or polypharmacy featured [35]. These findings emphasise the importance of proactive enquiry about OTC/POM use, medicine reviews, health and wellbeing checks (including liver function monitoring where paracetamol-containing products are misused), provision of take-home naloxone and medicine-safety counselling at the point of sale or supply.

The participants’ reports of iatrogenic dependence, especially among younger employed women using OTC codeine products, highlight opportunities for pharmacists to intervene earlier through structured advice, referral pathways, and deprescribing support. The need to increase awareness of OTC/POM misuse has also been suggested by Coombes and Cooper [16].

The participants’ perceptions of differences between people who misused OTC/POM alongside illicit substances and those who primarily misused legally sourced medicines are consistent with previous work, suggesting that differing psychosocial profiles and treatment needs may exist [36,37]. For pharmacists, this distinction is especially relevant: individuals who misuse only OTC/POM may be more likely to present in pharmacy settings and may require sensitive, non-stigmatising engagement to encourage disclosure and referral.

The participants’ mixed views on dedicated pathways for this group reflect wider debates about service design and require further consideration [16]. While separate pathways may improve engagement, they require appropriate resourcing and risk reinforcing stigma and fragmenting care [38]. The need for separate SMS/bespoke interventions should be determined by the needs of the individual: pharmacy-inclusive, multidisciplinary models may offer a more balanced approach and may be advantageous when there is refusal to access usual SMS [5,39].

The participants described significant challenges arising from unmanaged health conditions, long waiting times, and reluctance from other services to treat individuals with co-existing substance use. These findings highlight the need for integrated pharmacy–SMS collaboration to ensure timely access to pain management, mental health support, and medicine review.

Withdrawal symptoms were a major driver of continued misuse, and had an associated gateway effect, as predisposed individuals sourced illicit substances, unregulated supplies or larger amounts of combination codeine products, increasing risk [40]. Similarly, as people reportedly misused OTC/POM when unable to source their usual substance of choice [41], or to manage self-detoxification from them, this further highlights the need for appropriate withdrawal symptom management. This also reinforces the importance of pharmacist involvement in safe tapering, monitoring, and shared decision-making [7].

Concerns about prescribing practices, inconsistent pathways, and limited training emphasise the need for more specific guidance on managing problematic OTC/POM misuse [16,37]. Having a more confident and competent workforce may assist with recruitment and retention, in keeping with current national drivers and government strategy [12,19]. Retaining staff should optimise continuity of care and SMS engagement by enabling the same staff to provide support to the same individuals for the duration of their recovery journey. Improvements in SMS education and training provision, peer support and structured practice/clinical supervision should further develop collaborative and cross-boundary working [18,19,20].

The inclusion of staff with diverse roles strengthens the breadth of perspectives. No participants were accompanied, and the detail and openness in their responses (including those who chose to disclose being in recovery) indicated that the interviewer built a good level of rapport, and suggests that the anonymisation, piloting of associated documents and use of an interview guide were effective, as has been found in other studies [10]. The use of one interviewer limited variability in the way that interviews were conducted, and the independent review of the themes made the analysis process more robust.

Several limitations should be considered when interpreting these findings. For example, the participants were recruited from services delivered by a single third-sector provider, which may limit transferability to other provider models. Interviews were relatively brief (mean 18 min; range 7–40 min): this is partly explained by the duration not including any of the standardised interview introduction/closing remarks, which did not contain any information that would be useful in the analyses. While the participants provided rich accounts and data saturation was considered achieved, shorter interviews may have limited the depth of exploration. Additionally, data collection occurred during the COVID-19 pandemic, which may have influenced both service delivery and participant perspectives. The interviewer was employed within the same organisation, which may have influenced participant responses despite assurances of confidentiality and the absence of direct managerial relationships. Whilst no differences were identified when transcripts were independently reviewed, repeat interviews and member-checking may have improved assurance in the findings. An additional limitation was that potential participant numbers were not ascertained: this was difficult given the staff turnover during the time the research was conducted, complicated, for example, by COVID-19-related staff absences.

The participants also highlighted concerns that the term ‘SMS’ may discourage engagement among some individuals who misuse OTC/POM but do not identify with illicit substance use. Therefore, future research should explore how service naming, messaging, and accessibility influence help-seeking behaviour and whether alternative models of service delivery improve engagement. Quantitative research should also be considered to determine the prevalence of the challenges identified in this study and to evaluate their impact on treatment outcomes, service utilisation, and health policy.

Given likely ongoing changes to OTC/POM availability [42], future research should also explore if there are changes in experiences over time. As some staff disclosed that they were in recovery, this should further support a co-production approach to SMS developments [6,43]; however, considering how their experiences can better influence service improvements, this must be balanced with sensitivity to their desire for confidentiality [44]. Future research which formally captures whether the participant has lived experience, and their demographic characteristics, such as their age and ethnicity, may allow for comparisons in how their views and experiences may vary. Additionally, a comparison between the views of those working in SMS and other sectors (such as community pharmacy) should be considered, as roles and contexts differ, and this may enable the identification of how services may be further developed to improve care delivery for people who misuse OTC/POM.

## 5. Conclusions

This qualitative study explored the experiences of third-sector staff working in English community SMS who support adults affected by OTC/POM misuse. The participants predominantly described dependence on codeine-containing products, benzodiazepines, and gabapentinoids taken orally, alongside challenges related to withdrawal, treatment pathways, polypharmacy, and limited awareness of potential harms.

The findings suggest opportunities to strengthen routine enquiry about OTC/POM use; improve harm-reduction approaches; allow access to evidence-based tapering guidance and training; tailor psychosocial and pharmacological interventions; and enhance collaboration between pharmacy and SMS. The participants also identified potential differences in treatment needs among people presenting with OTC/POM misuse, although further research is needed to explore these observations.

Future studies should examine these issues quantitatively; evaluate service models for this population; and determine how pharmacy services can best contribute to prevention, early identification, and support for recovery.

## Figures and Tables

**Table 1:** Duration and method of participant interviews.

Participant ID	Interview Method	Duration (Hours: Minutes: Seconds)
001	Phone	00:40:15
002	Phone	00:20:23
003	Face to face	00:18:18
004	Face to face	00:11:08
005	Face to face	00:11:52
006	Phone	00:10:51
007	Phone	00:29:54
008	Face to face	00:07:19
009	Face to face	00:12:27
010	Face to face	00:09:25
011	Phone	00:12:32
012	Phone	00:14:43
013	Phone	00:29:17
014	Phone	00:23:04
015	Phone	00:22:06
016	Phone	00:28:01
017	Phone	00:32.10
018	Phone	00:17:18
019	Phone	00:15:02
020	MS Teams	00:15:28

## Data Availability

The raw data supporting the conclusions of this article will be made available by the authors upon request.

## Abbreviations

The following abbreviations are used in this manuscript:

OTC	Over the counter
POM	Prescription-only medication
SMS	Substance misuse services
